# Quantifying the Acute Care Costs of Neonatal Bacterial Sepsis and Meningitis in Mozambique and South Africa

**DOI:** 10.1093/cid/ciab815

**Published:** 2021-11-02

**Authors:** Céline Aerts, Shannon Leahy, Humberto Mucasse, Sanjay Lala, Justina Bramugy, Cally J Tann, Shabir A Madhi, Azucena Bardají, Quique Bassat, Ziyaad Dangor, Joy E Lawn, Mark Jit, Simon R Procter

**Affiliations:** 1 ISGlobal, Hospital Clínic, Universitat de Barcelona, Barcelona, Spain; 2 Paediatric Education and Research Ladder, Department of Paediatrics and Child Health, Faculty of Health Sciences, University of the Witwatersrand, Johannesburg, South Africa; 3 Centro de Investigação em Saúde de Manhiça, Maputo, Mozambique; 4 Department of Infectious Disease Epidemiology, Faculty of Epidemiology and Population Health, London School of Hygiene and Tropical Medicine, London, United Kingdom; 5 Maternal, Adolescent, Reproductive and Child Health Centre, London School of Hygiene and Tropical Medicine, London, United Kingdom; 6 Neonatal Medicine, University College London Hospitals, London, United Kingdom; 7 South African Medical Research Council: Vaccines and Infectious Diseases Analytics Research Unit, Faculty of the Health Sciences, University of the Witwatersrand, Johannesburg, South Africa; 8 Institució Catalana de Recerca i Estudis Avançats (ICREA), Barcelona, Spain; 9 Pediatrics Department, Hospital Sant Joan de Déu, Universitat de Barcelona, Esplugues, Barcelona, Spain; 10 Consorcio de Investigación Biomédica en Red de Epidemiología y Salud Pública, Madrid, Spain

**Keywords:** sepsis, meningitis, neonate, costs, health economics

## Abstract

**Background:**

Sepsis and meningitis are among the leading causes of neonatal deaths in sub-Saharan Africa (SSA). Neonatal sepsis caused ~400 000 deaths globally in 2015, half occurring in Africa. Despite this, there are few published data on the acute costs of neonatal sepsis or meningitis, with none in SSA.

**Methods:**

We enrolled neonates admitted to 2 hospitals in South Africa and Mozambique between 16 April 2020 and 1 April 2021. In South Africa all cases were microbiologically confirmed, but in Mozambique both clinically suspected and microbiologically confirmed cases were included. Data were collected on healthcare resource use and length of stay, along with information on household expenditure and caregiving. We used unit costs of healthcare resources in local currencies to estimate healthcare provider costs per patient and costs per household. Results were converted to 2019 international dollars (I$).

**Results:**

We enrolled 11 neonates in Mozambique and 18 neonates in South Africa. Mean length of stay was 10 days (median, 9 [interquartile range {IQR}, 4–14) and 16 days (median, 15 [IQR, 13–18]), respectively. In Mozambique we estimated mean household costs of I$49.62 (median, 10.19 [IQR, 5.10–95.12]) and hospitalization costs of I$307.58 (median, 275.12 [IQR, 149.43–386.12]). In South Africa these costs were I$52.31 (median, 30.82 [IQR, 19.25–73.08]) and I$684.06 (median, 653.62 [IQR, 543.33–827.53]), respectively.

**Conclusions:**

We found substantial costs associated with acute neonatal bacterial (all-cause) sepsis and meningitis in SSA. Our estimates will inform economic evaluations of interventions to prevent neonatal invasive bacterial infections.

**KEY FINDINGS**

**1. WHAT WAS KNOWN AND WHAT IS NEW?**

Globally, the few published studies on acute healthcare costs of neonatal sepsis and/or meningitis are from high-income countries, and mainly from the United States. There are no studies from sub-Saharan Africa despite the high disease burden in this region. Our study provides estimates of health care and household costs associated with acute neonatal sepsis and meningitis hospitalisations in Mozambique and South Africa.

**2. WHAT DID WE DO AND WHAT DID WE FIND?**

Using hospital records and discharge questionnaires of 29 neonates hospitalized in Mozambique and South Africa, we estimated mean costs to households and healthcare providers to be I$49.62 (median=10.19, IQR=5.10-95.12) and I$307.58 (median=275.12; IQR=149.43-386.12), and I$52.31 (median=30.82; IQR=19.25-73.08) and I$684.06 (median=653.62; IQR=543.33-827.53), respectively in 2019 I$.

**3. WHAT TO DO NOW IN PROGRAMMES?**

Neonatal complications including infections are an important cost to health systems and families, and are an important consideration for inclusion in financial protection schemes. Interventions to prevent neonatal sepsis and meningitis could reduce the costs to healthcare providers and households, as well as improving health outcomes.

**4. WHAT NEXT IN RESEARCH?**

Our results could be used in future research on the cost-effectiveness of interventions to prevent neonatal bacterial infections. Further costing studies with larger study populations, across a range of hospital settings and countries, and using similar tools to ours would make findings more generalisable and give insights into cost drivers and contextual differences.

Although child mortality has halved in the last 2 decades, deaths in the neonatal period accounted for a larger proportion, approximately 47% (2.4 million) of all under-5 deaths in 2019 [1]. The highest neonatal mortality rate is in the sub-Saharan Africa (SSA) region (United Nations), with sepsis and meningitis among the leading causes of neonatal deaths [2]. Neonatal sepsis, due to pathogens including group B *Streptococcus* (GBS), led to about 400 000 deaths globally in 2015, half of which occurred in the African region [3].

Despite this, there are few published studies on the cost of acute healthcare for sepsis and/or meningitis in neonates from low- and middle-income countries (LMICs), with data concentrated mainly in the United States (US) [4]. Furthermore, no studies have reported the costs borne by households, including indirect costs of care, thereby leading to a potential underestimation of the true societal costs [4]. Knowledge of the economic consequences of sepsis and/or meningitis is essential for economic evaluations that inform policy-making to prevent invasive bacterial infections in neonates [5].

The study was conducted both in Mozambique (categorized as a low-income country) and South Africa (categorized as an upper-middle-income country). In 2019, the gross domestic product per capita based on the purchasing power parity was I$1336 in Mozambique and I$13010 in South Africa [6].

## Objective

This article is part of a series on GBS worldwide. This paper addresses the lack of empirical data in LMICs, and SSA specifically, by estimating the costs to the healthcare system and households during acute episodes of bacterial sepsis and/or meningitis among neonates admitted to hospital in Mozambique and South Africa.

## METHODS

### Data Collection

This costing study was a linked component of a larger study on the outcomes after invasive GBS disease in young infants, for which the protocol has been published previously [7]. Data were collected among young infants (0–89 days of life) admitted for clinically suspected bacterial sepsis and/or meningitis. In South Africa all cases were confirmed microbiologically either by culture or detection by polymerase chain reaction (for meningitis cases) in a normally sterile site (blood or cerebrospinal fluid). In Mozambique cases were defined based on clinical suspected sepsis and/or meningitis at admission, with only some being microbiologically confirmed. Babies born with severe congenital abnormalities or culture positive results of organisms considered contaminants or skin commensals were excluded [7]. Furthermore, babies born below 32 weeks of gestational age were excluded as they may incur additional costs related to being born prematurely.

We enrolled all participants who met the inclusion criteria and who were admitted between 16 April 2020 and 1 April 2021 at the Chris Hani Baragwanath Academic Hospital, a tertiary-academic hospital situated in Soweto, South Africa; and the Manhiça District Hospital, the referral hospital for the district of Manhiça in Mozambique. The hospitals included in this study are public ones; healthcare is normally provided free of charge to all patients in Mozambique and to patients <6 years old in South Africa. Data collection took place during the middle of the severe acute respiratory syndrome coronavirus 2 pandemic, which prevented us from reaching the initial set sample size of 20 neonates per site.

Most participants were identified prospectively on admission. About 14% (4/29) of the neonates were admitted from birth. These were already in hospital and kept in due to developing sepsis and/or meningitis shortly after birth. Neonates who died shortly after admission were not excluded from the study. Data on hospital resource use were collected from medical records, including information on length of stay, drug use, diagnostic tests, and surgical procedures (Supplementary Materials, Tool 2). Unit costs for resources were provided by the hospitals in each study site, except for the cost per bed-day in Mozambique, which was taken from published literature [8]. To capture the economic impact of an infant’s hospitalization on households, a questionnaire was administered to the main caregiver at the time of discharge or soon after discharge, and included questions on out-of-pocket payments; medical, transportation, and accommodation expenses; and caregiving time (Supplementary Materials, Tool 1). Both hospital and household costs only refer to costs incurred during the length of neonatal hospital admission. If an infant died in hospital, no questionnaires were administered but data were still collected from hospital records. The final version of the questionnaire was first piloted before it was administered by trained local research team members. A consent form was signed by all neonates’ caregivers participating in the study.

### Analysis

We estimated household costs associated with the acute episode including direct and indirect costs, namely medical, transportation, and accommodation costs, as well as costs of informal caregiving. Healthcare provider costs were calculated based on the average length of stay, use of drugs, diagnostics, and supportive care, among others. Most unit costs were provided by the sites in local currencies for the year 2019. Wherever estimates were in another currency (ie, US dollars) and/or for another fiscal year, these were converted into the local currency using the appropriate exchange rate and/or inflated using the local consumer price index [9, 10]. To facilitate comparison between countries, estimates were converted into international dollars (I$) using the purchasing power parity conversion factor for 2019 [11]. Given that cost data are skewed, we report the median and interquartile range (IQR) alongside the mean costs. As a sensitivity analysis, we calculated costs for only microbiologically confirmed Mozambican cases. Data were analyzed in Stata and Excel.

**Ethical Considerations**

The overarching protocol for this multicountry observational study was granted ethical approval at the London School of Hygiene and Tropical Medicine (approval number 16246). Institutional review boards in each of the operating countries granted ethics approval (Mozambique approval number 98/CNBS/2019; South Africa approval number M190241), as well as the institutional review board of the World Health Organization (approval number ERC0.0003169). 

Data sharing agreements were jointly developed and signed by all collaborating partners. Data collection instruments used in this study are available in the Supplementary Materials.

## RESULTS

### Study Population

Overall, 29 neonates (18 from South Africa and 11 from Mozambique) were enrolled in the study population, which is summarized in Table 1 (the sensitivity analysis for Mozambique is summarized in Supplementary Table A1). The mean gestational age at birth was 38 weeks (median, 38 [IQR, 37–39]). The mean age on admission was 5 days (median, 2 [IQR, 1–7]) in Mozambique and 11 days (median, 9 [IQR, 6–13]) in South Africa. Among the study participants, 93% (27/29) had sepsis, while 7% (2/29) were diagnosed with meningitis. Ninety percent (26/29) of neonates were discharged alive; 2 were transferred to another hospital in Maputo, Mozambique (outcome unknown), and 1 South African neonate admitted at 4 days of age died after 19 days in hospital following diagnosis with sepsis (*Escherichia coli*).

**Table 1.** Descriptive Statistics of Neonatal Inpatients With Bacterial Sepsis and/or Meningitis Recruited Between 16 April 2020 and 1 April 2021

Characteristic	Mozambique	South Africa	Total
	(n = 11)	(n = 18)	(N = 29)
Sex			
Male	4 (36)	12 (67)	16 (55)
Female	7 (64)	6 (33)	13 (45)
Age, d			
0–6	7 (64)	6 (33)	13 (45)
7–14	4 (36)	8 (44)	12 (41)
15–28	0 (0)	4 (22)	4 (14)
Place of delivery			
Home	1 (9)	0 (0)	1 (3)
Hospital	10 (91)	18 (100)	28 (97)
Gestational age, wk			
32–34	0 (0)	2 (11)	2 (7)
35–37	4 (36)	6 (33)	10 (35)
>37	4 (36)	10 (56)	14 (48)
Unknown	3 (27)	0 (0)	3 (10)
Admitted at birth			
Yes	4 (36)	0 (0)	4 (14)
No	7 (64)	18 (100)	25 (86)
Birthweight, g			
<1500	1 (9)	0 (0)	1 (3)
1500–2500	4 (36)	5 (28)	9 (31)
2501–3000	1 (9)	7 (39)	8 (28)
>3000	5 (45)	6 (33)	11 (38)
Syndrome			
Meningitis	0 (0)	2 (11)	2 (7)
Sepsis	11 (100)	16 (89)	27 (93)
Bacteria detected			
Group B *Streptococcus*	1 (3)	8 (44)	9 (31)
*Escherichia coli*	0 (0)	6 (33)	6 (21)
*Staphylococcus aureus*	0 (0)	4 (22)	4 (14)
Gram-negative rod	5 (45)	0 (0)	5 (17)
Not microbiologically tested	5 (45)	0 (0)	5 (17)
Length of stay, d			
0–7	5 (45)	0 (0)	5 (17)
7–14	4 (36)	6 (33)	10 (34)
>15	2 (18)	12 (67)	14 (48)
Discharge status			
Discharged alive	9 (82)	17 (94)	26 (90)
Referred to another hospital	2 (18)	0 (0)	2 (7)
Died	0 (0)	1 (6)	1 (3)

Data are presented as No. (%). Percentages may not sum to 100% due to rounding.

### Cost Estimates

Hospitalization and household costs for the 2 countries are described in Table 2. For Mozambique, results of the sensitivity analysis including only microbiologically confirmed cases are provided in Supplementary Table A2.

**Table 2.** Descriptive Analysis of Cost Data for Neonatal Sepsis and/or Meningitis Inpatients (International Dollars 2019)

Hospitalization Costs	Mozambique (n = 11)			South Africa (n = 18)		
	Mean	Median	IQR	Mean	Median	IQR
Hospital stay (routine ward or ICU bed)	136.31	123.81	75.66–171.95	379.41	349.62	308.83–419.55
Supportive care						
Oral feeding	9.26	0.00	0.00–0.00	Included in the hospital daily rate		
Nasogastric feeding	NA	NA	NA	Included in the hospital daily rate		
Intravenous fluid	NA	NA	NA	Included in the hospital daily rate		
Parenteral feeding	NA	NA	NA	Included in the hospital daily rate		
Nasal cannula oxygen	NA	NA	NA	Included in the hospital daily rate		
Phototherapy	NA	NA	NA	Included in the hospital daily rate		
Hypothermia	Data not available					
Drug therapy						
Antibiotics (first line)	43.68	0.00	0.00–78.77	25.29	14.19	7.02–45.28
Antibiotics (second line)	3.78	0.00	0.00–0.00	14.04	2.93	0.00–17.26
Antibiotics (third line)	NA	NA	NA	0.89	0.00	0.00–0.00
Antifungals	NA	NA	NA	0.64	0.00	0.00–0.00
Anticonvulsants	NA	NA	NA	2.27	0.00	0.00–0.00
Other drugs^a^	0.46	0.46	0.00–0.00	2.45	0.00	0.00–5.02
Laboratory test						
Blood culture	22.75	0.00	0.00–41.71	19.20	16.08	16.08–24.12
Lumbar puncture	44.07	0.00	0.00–121.19	27.65	23.70	23.70–23.70
CSF culture	35.53	0.00	0.00–97.72	17.26	14.79	14.79–14.79
Urine culture	6.05	0.00	0.00–0.00	9.99	8.99	8.99–8.99
Full blood count test	2.72	0.00	0.00–0.00	18.32	18.32	9.16–27.48
Blood biochemistry	NA	NA	NA	81.00	36.45	36.45–100.24
Serum bilirubin test	NA	NA	NA	5.46	0.00	0.00–5.79
Blood glucose test	2.96	0.00	0.00–0.00	12.25	4.79	0.00–4.79
Diagnostic assessment						
Hearing assessment	NA	NA	NA	Included in the hospital daily rate		
Electrocardiogram	NA	NA	NA	Included in the hospital daily rate		
Diagnostic imaging						
Radiograph	NA	NA	NA	4.18	0.00	0.00–7.52
Ultrasound scan	NA	NA	NA	13.03	0.00	0.00–19.55
Echocardiogram	NA	NA	NA	50.13	0.00	0.00–0.00
Total	307.58	275.12	149.43–386.12	684.35	653.92	543.64–827.83
Household Expenses	Mozambique (n = 7)^b^			South Africa (n = 18)		
	Mean	Median	IQR	Mean	Median	IQR
Healthcare	40.04	0.00	0.00–67.94	10.03	0.00	0.00–0.00
Transportation	9.58	7.64	4.25–12.74	42.28	30.83	19.25–70.38
Food and accommodation	0.00	0.00	0.00–0.00	0.00	0.00	0.00–0.00
Total	49.62	10.19	5.10–95.12	52.31	30.82	19.25–73.08

Abbreviations: CSF, cerebrospinal fluid; ICU, intensive care unit; IQR, interquartile range; NA, not applicable.

^a^Based on 1 estimate.

^b^Less than 11 due to missing values in the cost of transportation.

#### Hospitalization Costs

The mean length of stay of 16 days (median, 15 [IQR, 13–18]) in South Africa was substantially longer than in Mozambique, which was 10 days (median, 9 [IQR, 4–14]), and 11 days (median, 10 [IQR, 7–14]) when including only microbiologically confirmed cases. The mean hospitalization cost per neonate of I$684.06 (median, 653.62 [IQR, 543.33–827.53]) in South Africa was also higher than the cost of I$307.58 (median, 275.12 [IQR, 149.43–386.12]) in Mozambique. However, for microbiologically confirmed cases this increased to I$413.41 (median, 386.12 [IQR, 221.48–514.39]), primarily due to higher laboratory costs.

#### Household Direct Costs

Most households incurred no direct healthcare expenses related to their child’s acute infection. The mean healthcare expenditure per patient was I$40.04 in Mozambique and I$10.03 in South Africa, but median costs were zero in both countries.

In both countries, food and accommodation at the hospital are provided to caregivers without charge, so the main direct costs were transportation expenses. On average, households spent I$9.58 in Mozambique and I$41.64 in South Africa on transportation. Therefore, the overall direct household costs were I$49.62 (median, 10.19 [IQR, 5.10–95.12]) and I$52.31 (median, 30.08 [IQR, 19.25–73.08]) in Mozambique and South Africa, respectively.

#### Household Costs of Informal Care (Indirect Costs)

In 97% of cases the caregiver accompanying the child to the hospital was the mother, and otherwise the father. Caregivers’ age ranged from 15 to 36 years. In Mozambique, caregivers reported spending 52 hours (median, 38.5 [IQR, 19–86]) on extra care. In South Africa, hours of informal care varied significantly between caregivers with a mean of 76 hours (median, 3.5 [IQR, 0–120]). Among caregivers, 79% did not earn an income, but all reported that none of the extra time spent caring for their child would have been normally spent in paid work.

## Discussion

The mean healthcare provider costs of treating neonatal sepsis and meningitis were substantially higher in South Africa than Mozambique: I$684.06 (median, 653.62 [IQR, 543.33–827.53]) vs I$307.58 (median, 275.12 [IQR, 149.43–386.12]), although this difference was smaller when including only microbiologically confirmed cases in Mozambique. Higher costs in South Africa can be partly explained by intercountry differences in resource unit costs, and by the shorter length of stay and more limited standard of care provided in Mozambique. However, the direct costs from the household perspective were more similar between the 2 countries. These were estimated to be I$49.62 (median, 10.19 [IQR, 5.10–19.25]) in Mozambique and I$52.31 (median, 30.82 [IQR, 19.25–73.08]) in South Africa. Such expenses were not considered catastrophic except for 2 households, 1 in each country, for whom these costs represented >40% of their monthly income [12]. Although the cost of informal caregiving could not be calculated for most caregivers as they did not earn an income, all caregivers reported that the extra hours that they spent with their child would not have been spent in paid work. This suggests zero financial opportunity cost for caring for the child, but also may reflect the poor communities from which these children come.

The lack of existing literature on this topic prevents any comparison with other studies in SSA. However, based on a few other LMIC studies, the hospitalization costs found in our study appear broadly consistent with previously reported cost of treating sepsis (2017 I$642) and meningitis (2017 I$198–I$8078) [4].

While our study is one of the first to report costs associated with bacterial sepsis and meningitis in SSA, it has some limitations. First, our analysis was only descriptive since the small sample size prevented us from investigating the determinants of costs and differences between countries. Second, we collected data from only 1 hospital in each country, which together with the sample size limits the generalizability of our findings. Third, in Mozambique our results include suspected, as well as microbiologically confirmed cases of sepsis, which may underestimate the true costs. Last, this study does not include the healthcare costs associated with the longer-term impact of the disease such as neurodevelopment deficit. Furthermore, this study does not include preterm infants because it would require a larger sample size and controlling for potential cofounders, which is beyond the scope of this study.

Despite these limitations, our multicountry study addresses an important gap in the literature that can inform policymaking. We found substantial costs associated with acute neonatal bacterial sepsis and meningitis in 2 countries in SSA, and our estimates will be useful to inform cost-effectiveness analyses of interventions to prevent invasive bacterial infections in neonates.

## Supplementary Data

Supplementary materials are available at *Clinical Infectious Diseases* online. Consisting of data provided by the authors to benefit the reader, the posted materials are not copyedited and are the sole responsibility of the authors, so questions or comments should be addressed to the corresponding author.

ciab815_suppl_Supplementary_Material

## Notes

***Author contributions.*** M. J., J. E. L., S. R. P., Z. D., and Q. B. conceptualized the study and developed the methodology. C. J. T. helped devise the data collection instrument. C. A., S. Le., H. M., S. La., J. B., and A. B. collected the data. C. A. conducted the analysis with input from S. R. P., Q. B., and Z. D. The manuscript was drafted by C. A. and S. R. P. All authors reviewed and helped to revise the manuscript and agreed the final version.

***Disclaimer.*** The funders had no role in study design, data collection and analysis, decision to publish, or preparation of the manuscript.

***Financial support.*** This work was supported by the Bill & Melinda Gates Foundation (grant number INV-009018 to the London School of Hygiene and Tropical Medicine [LSHTM], principal investigator [PI]: Joy Lawn). Centro de Investigação em Saúde de Manhiça (CISM) is supported by the government of Mozambique and the Spanish Agency for International Development. ISGlobal receives support from the Spanish Ministry of Science and Innovation through the Centro de Excelencia Severo Ochoa 2019–2023 program (CEX2018–000806-S) and support from the Generalitat de Catalunya through the Centres de Recerca de Catalunya program.

***Supplement sponsorship.*** This supplement is sponsored by Bill & Melinda Gates Foundation.

***Potential conflicts of interest.*** Many contributors to this supplement have received funding for their research from foundations, especially the Bill & Melinda Gates Foundation, the Meningitis Research Foundation, the European and Developing Countries Clinical Trials Partnership (EDCPT), and others. A. B. and J. B. report subawards from LSHTM to Mozambique and an EDCTP-funded fellowship (number 2836; PI: Marieke Van der Zalm). Q. B. reports subawards from LSHTM to ISGlobal and CISM/Fundação Manhiça, Mozambique. Z. D. and S. L. report subawards from LSHTM to Wits Health Consortium. M. J. reports being a scientific committee member of the 2nd International Symposium on *Streptococcus agalactiae* Disease. S. La reports subawards from LSHTM to the Wits Health Consortium, University of the Witwatersrand, Johannesburg, South Africa. H. M. reports subawards from LSHTM to Mozambique. S. A. M. has collaborated on group B *Streptococcus* (GBS) grants funded by GlaxoSmithKline and Pfizer and received personal fees for being a member of Bill & Melinda Gates Foundation advisory committee; he has also collaborated on a GBS grant funded by Minervax. All other authors report no potential conflicts of interest.
