# Supplementary material for: Quantifying the Acute Care Costs of Neonatal Bacterial Sepsis and Meningitis in Mozambique and South Africa
Source: Clin Infect Dis. 2021 Nov 2;74(Suppl 1):S64–9. doi: 10.1093/cid/ciab815 (PMC8776306; doi:10.1093/cid/ciab815)
Supplement: ciab815_suppl_Supplementary_Material [file ciab815_suppl_supplementary_material.docx]

**SUPPLEMENTARY MATERIAL**

**Supplement Title:** Every Country, Every woman, Every Child; Group B Streptococcal Disease Worldwide

**Paper Title:** Quantifying the acute care costs of neonatal bacterial sepsis and meningitis in Mozambique and South Africa

**Authors**: Céline Aerts^1*^, Shannon Leahy^2^, Humberto Mucasse^3^, Sanjay Lala^2^, Justina Bramugy^3^, Cally J. Tann^4,5,10^, Shabir A. Madhi^6^, AzucenaBardají^1,3^, Quique Bassat^1,3,7,8,9^, Ziyaad Dangor^2^, Joy E. Lawn^4,5^, Mark Jit^4^, Simon R. Procter^4,5^*

**Corresponding authors:** Simon Procter, Department of Infectious Disease Epidemiology, London School of Hygiene & Tropical Medicine, London, United Kingdom;

Email: [simon.procter@lshtm.ac.uk](mailto:simon.procter@lshtm.ac.uk);

Céline Aerts, ISGlobal, Hospital Clínic - Universitat de Barcelona, Barcelona, Spain;

Email: [celine.aerts24@gmail.com](mailto:celine.aerts24@gmail.com)

Affiliations

1. ISGlobal, Hospital Clínic – Universitat de Barcelona, Barcelona, Spain
2. Paediatric Education and Research Ladder, Department of Paediatrics and Child Health, Faculty of Health Sciences, University of the Witwatersrand, Johannesburg, South Africa
3. Centro de Investigação em Saúde de Manhiça (CISM), Maputo, Mozambique
4. Department of Infectious Disease Epidemiology, Faculty of Epidemiology and Population Health, London School of Hygiene & Tropical Medicine, London, United Kingdom
5. Maternal, Adolescent, Reproductive and Child Health (MARCH) Centre, London School of Hygiene & Tropical Medicine, London, United Kingdom
6. South African Medical Research Council: Vaccines and Infectious Diseases Analytics Research Unit, Faculty of the Health Sciences, University of the Witwatersrand, Johannesburg, South Africa
7. ICREA, Pg. Lluís Companys 23, 08010 Barcelona, Spain
8. Pediatrics Department, Hospital Sant Joan de Déu, Universitat de Barcelona, Esplugues, Barcelona, Spain
9. Consorcio de Investigación Biomédica en Red de Epidemiología y Salud Pública (CIBERESP), Madrid, Spain
10. Neonatal Medicine, University College London Hospitals, London UK

Table of Contents

[Supplementary Acute Data Collection Tool 1: Pre-discharge survey 3](#_Toc81487943)

[Supplementary Acute Data Collection Tool 2: Hospital Extraction Form 13](#_Toc81487944)

[Supplementary Acute Data Collection Tool 3: Hospital Health Unit Costs 29](#_Toc81487945)

[Supplementary Table A1: Descriptive statistics of the patients with bacterial sepsis and/or meningitis including only microbiological cases confirmed cases. 31](#_Toc81487946)

[Supplementary Table A2: Cost estimates (International Dollars 2019) including only microbiologically confirmed cases. 32](#_Toc81487947)

# Supplementary Acute Data Collection Tool 1: Pre-discharge survey

Pre-discharge questionnaire to assess family costs related to acute neonatal GBS disease (Generic)

| **Introduction** |  |
| --- | --- |
| Thank you again for your participation in the study, which aims to understand the health and financial needs of children who have neonatal sepsis when they are babies.  Your participation in our study is voluntary, and non-participation will not influence your health care or your relationship with your midwife, doctor or other health care professional. You can stop the interview at any point if you no longer want to be part of the study.  We have an information form about our study that I will read to you now and ask your consent for ongoing participation in this study.  INTERVIEWER READS INFORMATION AND CONSENT FORM, GIVES INFORMATION FORM TO CAREGIVER/MOTHER AND KEEPS CONSENT FORM. GO TO CONSENT SECTION TO SELECT CONSENT ‘YES’ OR ‘NO’ AS APPROPRIATE.  IF CONSENT YES, CONTINUE TO GBS QUESTIONNAIRE | |
|  | |

SECTION 1 – PARTICIPANT AND HOUSEHOLD DETAILS

**This section captures background information on the participant, main caregiver and household.**

Study information

| **ID** | **Question / Data Item** | **Response Options** | **Comments/ Filter** |
| --- | --- | --- | --- |
| 1.1 | Study Site |  | *(tick one appropriate answer)* |
| 1.2 | Country code | (01)  Argentina  (02)  India  (03)  Kenya  (04)  Mozambique  (05)  South Africa | *(tick one appropriate answer)* |
| 1.3 | Clinic/Hospital code |  | ***(indicate clinic code, interviewer ID and interview date correctly)*** |
| 1.4 | Interviewer ID |  |  |
| 1.5 | Interview Date | d  d  m  m  y  y  y  y |  |
|  | **Respondent Identification** |  |  |
| 1.6 | What is **YOUR** relationship to **THE CHILD**? | Mother  Father  Grandparent  Aunt / Uncle  Sibling  Other (biological relation)  Other (not biological relation) |  |
| 1.7 | Are you the main caregiver for this child? | YES  NO | If yes, skip to 2.1 |
| 1.8 | What is **the main caregiver** relationship to **THE CHILD**? | Mother  Father  Grandparent  Aunt / Uncle  Sibling  Other (biological relation)  Other (not biological relation) | main caregiver, which is the person from your household who usually spends the most time caring for THIS CHILD. |

Participant information

| **ID** | **Question / Data Item** | **Response Options** | **Comments** |
| --- | --- | --- | --- |
|  | **Child Identification** |  |  |
| 2.1 | Study Participant ID |  | ***Unique for the study and auto generated when participant list imported into database*** |
| 2.1a | HDSS Participant ID |  | *Only in sites with health and Demographic Surveillance System (HDSS)* |
| 2.1b | HDSS Household ID |  | *Only in sites with health and Demographic Surveillance System (HDSS)* |
| 2.2 | Participant Name | Surname ………………………….  First name……………………… | ***(indicate name correctly)*** |
| 2.3 | Participant Sex | Male  Female | *(tick one appropriate answer)* |
| 2.4 | Participant Age | Don’t Know | ***(indicate Age)***  ***(tick the box if don’t know)*** |
| 2.5 | Participant Date of Birth | d  d  m  m  y  y  y  y | ***(indicate date of birth)*** |
| 2.6 | Participant Ethnic Group | Black  Coloured  Other ethnic group (Specify)………………. | *(tick appropriate answer and specify where needed)* |
| 2.7 | Participant Religion | Christian  Muslim  Traditionalist  Atheist | *(tick one appropriate answer)* |
| 2.8 | Language | English  Zulu  Sesotho  Other(Specify)………………. | *(tick appropriate answer and specify where needed)* |

Main caregiver information or closest available person

**The following questions relate to the main caregiver, that is the person from your household who has spent the most time caring for THIS CHILD since they became ill. If the respondent is not the main caregiver, then please ask them to answer the remaining questions from the perspective of the main caregiver.**

| **ID** | **Question / Data Item** | **Response Options** | **Comments** |
| --- | --- | --- | --- |
| 3.1 | **RECORD** the sex of the **MAIN CAREGIVER** | Male  Female | *(tick one appropriate answer)* |
| 3.2 | How old are **YOU/MAIN CAREGIVER**? | Don’t Know | ***(indicate Age)***  ***(tick the box if don’t know)*** |
| 3.3 | Can **YOU/MAIN CAREGIVER** read or write a simple sentence? | YES  NO | *(tick one appropriate answer)* |
| 3.4 | How many years did **YOU/MAIN CAREGIVER** spend in formal education? | Don’t Know | ***(indicate years)***  ***(tick the box if don’t know)*** |
| 3.5 | What is **YOU/MAIN CAREGIVER** highest level of education? | No formal  Early childhood education  Primary  Secondary  College/technical training  University | *(tick one appropriate answer)* |
| 3.6 | Are **YOU/MAIN CAREGIVER** the main income earner of the household? | YES  NO |  |
| 3.7 | What is **YOUR/MAIN CAREGIVER** occupation? | Working (formal/informal employment)  Seeking work  Homemakers  Students  Others (Specify)_______________ | *(tick one appropriate answer)* |
| 3.8 | What is **YOUR/MAIN CAREGIVER** current work status? | Income from paid employment  Income from self-employment  Unpaid work  Housework (including caring responsibilities)  Retired  Other | **If unpaid / housework / retired / other skip to 7.1** |
| 3.9 | On average how much do **YOU/MAIN CAREGIVER** earn per hour from paid work or self-employment? | Local currency    Don’t know | ***(indicate amount)***  ***(tick the box if don’t know)*** |
| 3.10 | On average how many hours paid work or self-employment do **YOU/MAIN CAREGIVER** work per week? | H  H  M  M  Don’t know | ***(indicate hours, if they don’t work put zero)***  ***(tick the box if don’t know)*** |

Household information

| **ID** | **Question / Data Item** | **Response Options** | **Comments** |
| --- | --- | --- | --- |
|  | **Household location** |  |  |
| 4.1 | Location of household (name of village, town, district) | Location name  ……………………………………… |  |
| 4.2 | Location type? | Urban  Traditional  Farm | *(tick one appropriate answer)* |
|  | **Household Occupants** |  |  |
| 5.1 | How many people in total live in the household? | Don’t Know | ***(indicate whole number)***  ***(tick the box if don’t know)*** |
| 5.2 | How many of these people are children aged under 18? | Don’t Know | ***(indicate whole number)***  ***(tick the box if don’t know)*** |
| 5.3 | How many of these people are adults over the age of 60? | Don’t Know | ***(indicate whole number)***  ***(tick the box if don’t know)*** |
| 5.4 | Have any children from the household ever been given up for adoption? | YES  NO | *Including informal adoption.* |
|  | **Socio-Economic Status** |  |  |
| 6.1 | Add country-specific asset index questions or input for pre-existing wealth index value from DHS here | |  |
|  | **Household Income** |  |  |
|  | What is the average **MONTHLY** income of your household from each of the following sources? | |  |
| 7.1 | What is the average total monthly income that you receive from all sources (i.e., total after any deductions)? | Local currency    Don’t know | ***(indicate income)***  ***(tick the box if don’t know)***  **If DON’T KNOW, SKIP to 12.1** |
| 7.2 | Income after tax from employment by adults living in your household? | Local currency    Don’t know | ***(indicate income)***  ***(tick the box if don’t know)*** |
| 7.3 | Income after tax from paid employment by children of school age living in your household? | Local currency    Don’t know | ***(indicate income)***  ***(tick the box if don’t know)*** |
| 7.4 | Income received from pensions by members of your household? | Local currency  Don’t know | ***(indicate income)***  ***(tick the box if don’t know)*** |
| 7.5 | Income received from social welfare, cash-transfers or other government payments? | Local currency    Don’t know | ***(indicate income)***  ***(tick the box if don’t know)*** |
| 7.6 | What is the estimated market value of food consumed by your household each **MONTH** that you produce yourselves? | Local currency  Don’t know | Aim is to estimate value of production. |
| 7.7 | What is the estimated market value clothes produced by your household each **MONTH** that you use yourselves? | Local currency    Don’t know | Aim is to estimate value of production. |
| 7.8 | Did your total household income decrease as a result of caring for **your child**? (Consider changes to all income including paid work and benefit payments.) | YES  NO | ***(Note for data collector: it should be made clear that this should be in reference to the before and after care for this particular child)*** |

Household expenditure

| **ID** | **Question / Data Item** | **Response Options** | **Comments** |
| --- | --- | --- | --- |
|  | **Total Expenditure** |  |  |
| 8.1 | Over the past **MONTH** what was your estimated total household expenditure? | Local currency  Don’t know | ***(indicate expenditure)***  ***(tick the box if don’t know)*** |
|  | **Expenditure by Category** |  |  |
| 9.1 | Over the past **MONTH** how much has your household spent in total on healthcare?  (e.g. hospital or clinic fees, doctor’s fees, traditional healers, medical tests, medications, assistive devices such as spectacles, hearing aids, walking canes) | Local currency    Don’t know | ***(indicate amount)***  ***(tick the box if don’t know)*** |
| 9.2 | Over the past **MONTH** how much has your household spent on transportation?  (e.g. Taxi / bus / train fares, petrol / oil, vehicle maintenance, parking fees, air travel) | Local currency  Don’t know | ***(indicate amount)***  ***(tick the box if don’t know)*** |
| 9.3 | Over the past **MONTH** how much has your household spent on education?  (e.g. school / tuition / university fees, tutoring or extra classes, school material such as books and uniforms) | Local currency    Don’t know | ***(indicate amount)***  ***(tick the box if don’t know)*** |
| 9.4 | Over the past **MONTH** how much has your household spent on food? | Local currency    Don’t know | ***(indicate amount)***  ***(tick the box if don’t know)*** |
| 9.5 | Over the past **MONTH** what is the estimated value of food your household has consumed that you received as gifts or produced yourselves? | Local currency  Don’t know | ***(indicate amount)***  ***(tick the box if don’t know)*** |
|  | **Health Insurance** |  |  |
| 10.1 | Are the members of your household covered by any form of private or government health insurance/medical aid scheme? | No insurance  Government insurance/NHI  Private Insurance  Others (specify) | **If “no insurance” skip to section 2.** |
| 10.2 | If you have healthcare insurance how much of your household healthcare expenditure from the past **MONTH** was or will be reimbursed/covered directly by the insurer? | Local currency    Don’t know | ***(indicate amount)***  ***(tick the box if don’t know)***  ***GO TO SECTION 2*** |

SECTION 2 – COSTS RELATED TO ACUTE INFECTION

Acute healthcare costs

The following questions relate to the cost of healthcare for **your child** from the time they became ill until the time of discharge.

| **ID** | **Question / Data Item** | **Response Options** | **Comments** |
| --- | --- | --- | --- |
|  | **Healthcare costs** |  |  |
| 11.1 | In total how much has your household spent on healthcare for **your child** since they became ill?  (e.g. hospital fees, doctor’s fees, medical tests, medications) | Local currency    Don’t know | ***(indicate amount)***  ***(tick the box if don’t know)*** |
| 11.2 | How much of this was or will be paid or refunded by public or private health insurance? | Local currency    Don’t know | ***(indicate amount)***  ***(tick the box if don’t know)*** |
| 11.3 | In total, how many days did **your child** spend in hospital? | Don’t Know | ***(indicate number of days)***  ***(tick the box if don’t know)*** |
| 11.4 | How many trips did **you / caregiver name** or someone else in your household travel to the hospital while **your child** was ill? | Don’t Know | ***(indicate number of trips)***  ***(tick the box if don’t know)*** |
| 11.5 | How far did you/they have to travel to the hospital? | Don’t know | ***(indicate number kilometre)***  ***(tick the box if don’t know)*** |
| 11.6 | In total how much did your household spend on travelling to the hospital?  e.g. bus or taxi fares, cost of petrol, parking fees. | Local currency    Don’t know | ***(indicate amount)***  ***(tick the box if don’t know)*** |
| 11.7 | Did **you / caregiver name** or someone else from your household stay with **your child** while they were in hospital? | YES  NO | **If no skip to 12.1a** |
| 11.8 | If so, how many days did they stay? | Don’t Know | ***(indicate number of days)***  ***(tick the box if don’t know)*** |
| 11.9 | In total how much did your household spend on food and accommodation when visiting **your child**? | Local currency    Don’t know | ***(indicate amount)***  ***(tick the box if don’t know)*** |

Informal caregiving

The following questions relate to the time spent caring for **your child** from the time they became ill until the time of discharge.

| **ID** | **Question / Data Item** | **Response Options** | **Comments** |
| --- | --- | --- | --- |
|  | **Informal caregiving** |  |  |
| 12.1a | Since **your child** became ill how many **extra days** have **you / caregiver name** spent providing informal care to them because of their illness?  e.g. time spent staying with them in hospital when you would not normally be caregiving. | Days  Don’t Know | ***(indicate number of EXTRA days on which care was provided)***  ***(tick the box if don’t know)*** |
| 12.1b | On average how much **extra** time have **you / caregiver name** spent **per day** providing informal care to them because of their illness? | Per day  H  H  M  M  Don’t know | ***(indicate the AVERAGE number of EXTRA hours per day)***  ***(tick the box if don’t know)*** |
| 12.1.c | In total how much **extra** time have **you / caregiver name** spent **per** providing informal care to them because of their illness? | Total  H  H  M  M  Don’t know | ***(indicate the total number of extra hours 12.1a x 12.1b )***  ***(tick the box if don’t know)*** |
| 12.2 | In total how much of this extra time would have normally been spent in paid work? | H  H  M  M  Don’t know | ***(indicate the number of hours)***  ***(tick the box if don’t know)*** |
|  | **Valuation** |  |  |
| 13.1 | Imagine that **your child** needs **one extra hour of informal care** per week and that government pays you for lending this extra hour of informal care.    What is the **lowest** amount you would be willing to accept to provide the extra hour of informal care? | I do not want to receive financial compensation for providing an extra hour of informal care.  The lowest amount I would be willing to accept is:  Local currency    I do not want to provide informal care for an extra hour regardless of how much the government might pay me. | ***(indicate amount)***  ***(tick the box if don’t know)*** |

Costs of coping

| **ID** | **Question / Data Item** | **Response Options** | **Comments** |
| --- | --- | --- | --- |
|  | **Borrowing** |  |  |
| 14.1 | Has your household sold, or will you need to sell, any property to cover the costs of healthcare for **your child**? | YES, already borrow  YES, plan to borrow  NO | *(tick one appropriate answer)*  **If no skip to 15.1** |
| 14.2 | If yes, how much did you borrow, or do you plan to borrow? | Local currency    Don’t know | ***(indicate amount)***  ***(tick the box if don’t know)*** |
| 14.3 | From whom did you, or do you plan to, borrow? | Family  Friends  Private bank  Cooperative  Informal money lender  Other | *(tick answer, you can tick more than one choice)* |
| 14.4 | What was the interest rate on the loan, or what will it be? | Specify interest rate…………….  No interest  Not expected to repay the loan.  Don’t know | **If “not expected to repay the loan” then skip to 15.1** |
| 14.5 | How long will it take to repay the loan? | (years)  (months)  Don’t know | Specify total loan length in years and months. |
|  | **Asset Sales** |  |  |
| 15.1 | Has your household sold, or will you need to sell, any property to cover the costs of healthcare for **your child**? | YES  NO | *(tick one appropriate answer)*  **If no skip to 16.1** |
| 15.2 | If yes, what property did you sell, or do you plan to sell? | Land  Livestock  Vehicle  Household items  Farm produce  Other (specify) | *(tick answer, you can tick more than one choice)* |
| 15.3 | What is the estimated market value of the property that you sold or plan to sell? | Local currency    Don’t know | ***(indicate amount)***  ***(tick the box if don’t know)*** |
| 15.4 | How much did you earn from the sale of your property? | Local currency    Don’t know | ***(indicate amount)***  ***(tick the box if don’t know)*** |
| 15.5 | Do you expect the sale of your property to reduce your household income or reduce the amount of food or clothing that your household is able to produce? | YES  NO | *(tick appropriate answer)*  **If no skip to 16.1** |
| 15.6 | How much did, or do you expect your household income to reduce per **MONTH**? | Local currency    Don’t know | ***(indicate amount)***  ***(tick the box if don’t know)*** |
| 15.7 | What is the estimated market value of the food or clothing that your household is no longer able to produce each **MONTH**? | Local currency    Don’t know | ***(indicate amount)***  ***(tick the box if don’t know)*** |
|  | **Other Coping Mechanisms** |  |  |
| 16.1 | Have any children in your household dropped out of education, or do you expect them to drop out of education, to help cover the costs of healthcare for **your child**? | YES, already dropped out  YES, plan to drop out  NO | *(tick appropriate answer)*  **If no skip to 16.3** |
| 16.2 | If so, how many children? |  | ***(indicate number)*** |
| 16.3 | Has anyone in your household taken on extra work, or does anyone plan to take on extra work, to help cover the costs of healthcare for **your child**? | YES, have taken on extra work  YES, plan to take on extra work  NO | *(tick appropriate answer)* |

**End of QUESTIONNAIRE**

# Supplementary Acute Data Collection Tool 2: Hospital Extraction Form

Hospital healthcare resource extraction tool to assess economic impact of acute neonatal GBS disease

This document sets out the data on costs of acute hospital care that to be extracted from patient notes for each participant. A separate questionnaire is used to capture the costs and economic impact from the household perspective.

SECTION 1 – STUDY AND PARTICIPANT BACKGROUND

| **ID** | **Question / Data Item** | **Response Options** | **Comments** |
| --- | --- | --- | --- |
|  | **Study information** |  |  |
| 1.1 | Study Site |  | *(tick one appropriate answer)* |
| 1.2 | Country code | (01)  Argentina  (02)  India  (03)  Kenya  (04)  Mozambique  (05)  South Africa | *(tick one appropriate answer)* |
| 1.3 | Clinic/Hospital code |  | ***(indicate clinic code, interviewer ID and data extraction date correctly)*** |
| 1.4 | Researcher ID |  |  |
| 1.5 | Date data extracted | d  d  m  m  y  y  y  y |  |
|  | **Participant identification** |  |  |
| 2.1 | Participant ID |  | ***Unique for the study and auto generated when participant list imported into database*** |
| 2.1a | HDSS Participant ID |  | *Only in sites with health and Demographic Surveillance System (HDSS)* |
| 2.1b | HDSS Household ID |  | *Only in sites with health and Demographic Surveillance System (HDSS)* |
| 2.2 | Participant Name | Surname ………………………….  First name……………………… | ***(indicate name correctly)*** |
| 2.3 | Participant Sex | Male  Female | *(tick one appropriate answer)* |
| 2.4 | Participant Age | Days  Don’t Know | ***(indicate Age in days)***  ***(tick the box if don’t know)*** |
| 2.5 | Participant Date of Birth | d  d  m  m  y  y  y  y | ***(indicate participant’s date of birth)*** |
|  | **Maternal information** | |  |
| 3.1 | Mother’s date of birth | d  d  m  m  y  y  y  y | ***(indicate mother’s date of birth)*** |
| 3.2 | Mother’s age | Years  Don’t know | ***(indicate mother’s age in years)*** |
| 3.3 | Mother’s HIV status | Negative  Positive  Don’t know | *(tick one appropriate answer)* |
|  | **Participant medical and birth history** | |  |
| 4.1 | Gestational age at birth | Numerical value (weeks)  Don’t Know | ***(indicate gestational age at birth in weeks)*** |
| 4.2 | Term / preterm | 37+ weeks  32-36 weeks  28-31 weeks  <28 weeks | *(tick one appropriate answer)* |
| 4.3 | Birth weight | Numerical value (grams) | ***(indicate birth weight in grams)*** |
| 4.4 | HIV status | Negative  Positive  Don’t know | *(tick one appropriate answer)* |
| 4.5 | Place of delivery | Home  Hospital  On the way to hospital | *(tick one appropriate answer)* |
| 4.6 | Mode of delivery | Vaginal birth  Assisted vaginal birth  Emergency caesarean section  Elective caesarean section | *(tick one appropriate answer)* |
| 4.7 | Did mother receive antibiotics? | No  Yes, intra-partum  Yes, post-partum | *(tick one appropriate answer)* |
|  | **Admission and Discharge Information** | |  |
| 5.1 | Date of admission | d  d  m  m  y  y  y  y | ***(indicate date of admission)*** |
| 5.2 | Was participant admitted from birth? | YES  NO | *(tick one appropriate answer)*  **If no skip to 5.4** |
| 5.3 | If yes, age at symptom onset (i.e. suspected infection)? | Days  Don’t Know | ***(indicate age in days at onset of symptoms)*** |
| 5.4 | If, no age at admission? | Numerical (days) |  |
| 5.5 | Date of discharge? | d  d  m  m  y  y  y  y |  |
| 5.6 | Status at discharge? | Discharged  Referral for control visits  Referral to another hospital  Dead | *(tick one appropriate answer)*  **If alive skip to 6.1** |
| 5.7 | Date of death? | d  d  m  m  y  y  y  y | ***(indicate date of death)*** |
|  | **Diagnosis** | |  |
| 6.1 | Pathogen | Group B streptococcus  Escherichia coli  Staphylococcus aureus  Coagulase negative staph.  Klebsiella  Listeria  Streptococcus pneumoniae  Group A streptococcus  Haemophilus influenzae  Other, please specific ____________________ | *(tick appropriate answer and specify where needed)* |
| 6.2 | Early or late onset? | Early onset (0 – 6 days old)  Late onset (7 – 89 days old) | *(tick one appropriate answer)* |
| 6.3 | Which of the following diagnoses have been confirmed? |  |  |
| 6.4 | Meningitis? | YES  NO | *(tick one appropriate answer)* |
| 6.5 | Sepsis? | YES  NO | *(tick one appropriate answer)* |
| 6.6 | Pneumonia? | YES  NO | *(tick one appropriate answer)* |
| 6.7 | Cellulitis / pustular skin infection? | YES  NO | *(tick one appropriate answer)* |
| 6.8 | Endocarditis? | YES  NO | *(tick one appropriate answer)* |
| 6.9 | Osteomyelitis / joint infection? | YES  NO | *(tick one appropriate answer)* |
| 6.10 | Abscess? | YES  NO | *(tick one appropriate answer)* |
| 6.11 | Seizures? | YES  NO | *(tick one appropriate answer)* |
| 6.12 | Subdural haematoma? | YES  NO | *(tick one appropriate answer)* |
| 6.13 | Hydrocephalus? | YES  NO | *(tick one appropriate answer)* |
| 6.14 | Severe jaundice? | YES  NO | *(tick one appropriate answer)* |
| 6.15 | Neonatal encephalopathy? | YES  NO | *(tick one appropriate answer)* |

SECTION 2 – HEALTHCARE RESOURCES

| **ID** | **Question / Data Item** | | **Response Options** | **Comments** |
| --- | --- | --- | --- | --- |
|  | **Length of Stay** |  | |  |
| 7.1 | Total length of admission (number of days)? | Days    Don’t know | |  |
| 7.2 | Number of days admitted to a paediatric/neonatal intensive care bed? (e.g. intubated on life-support) | Days    Don’t know | | Bed type may need local definition, this might be based on nursing ratio for different bed types. |
| 7.3 | Number of days admitted to a high-dependency care bed? (e.g. respiratory support / CPAP) | Days    Don’t know | | Bed type may need local definition, this might be based on nursing ratio for different bed types. |
| 7.4 | Number of days admitted to a special care bed? | Days    Don’t know | | Bed type may need local definition, this might be based on nursing ratio for different bed types. |
| 7.5 | Number of days admitted to a paediatric care bed? | Days    Don’t know | | Bed type may need local definition, this might be based on nursing ratio for different bed types. |
|  | **Supportive Care** |  | |  |
| 8.1 | Was oral feeding given? | Yes  No (none noted)  Unclear from notes  If yes, for how many days?  Days    Don’t know | | *(tick one appropriate answer, if yes then indicate number of days or tick don’t know if this is not known.)* |
| 8.2 | Was NG feeding given? | Yes  No (none noted)  Unclear from notes  If yes, for how many days?  Days    Don’t know | | *(tick one appropriate answer, if yes then indicate number of days or tick don’t know if this is not known.)* |
| 8.3 | Was IV fluid given? | Yes  No (none noted)  Unclear from notes  If yes, for how many days?  Days    Don’t know | | *(tick one appropriate answer, if yes then indicate number of days or tick don’t know if this is not known.)* |
| 8.4 | Was parenteral feeding given? | Yes  No (none noted)  Unclear from notes  If yes, for how many days?  Days    Don’t know | | *(tick one appropriate answer, if yes then indicate number of days or tick don’t know if this is not known.)* |
| 8.5 | Was nasal cannula oxygen given? | Yes  No (none noted)  Unclear from notes  If yes, for how many days?  Days    Don’t know | | *(tick one appropriate answer, if yes then indicate number of days or tick don’t know if this is not known.)* |
| 8.6 | Was high-flow / CPAP given? | Yes  No (none noted)  Unclear from notes  If yes, for how many days?  Days    Don’t know | | *(tick one appropriate answer, if yes then indicate number of days or tick don’t know if this is not known.)* |
| 8.7 | Was intubation and ventilation used? | Yes  No (none noted)  Unclear from notes  If yes, for how many days?  Days    Don’t know | | *(tick one appropriate answer, if yes then indicate number of days or tick don’t know if this is not known.)* |
| 8.8 | Was ECMO / bypass used? | Yes  No (none noted)  Unclear from notes  If yes, for how many days?  Days    Don’t know | | *(tick one appropriate answer, if yes then indicate number of days or tick don’t know if this is not known.)* |
| 8.9 | Was inhaled nitric oxide given? | Yes  No (none noted)  Unclear from notes  If yes, for how many days?  Days    Don’t know | | *(tick one appropriate answer, if yes then indicate number of days or tick don’t know if this is not known.)* |
| 8.10 | Was therapeutic hypothermia performed? | Yes  No (none noted)  Unclear from notes  If yes, for how many days?  Days    Don’t know | | *(tick one appropriate answer, if yes then indicate number of days or tick don’t know if this is not known.)* |
| 8.11 | Was phototherapy used? | Yes  No (none noted)  Unclear from notes  If yes, for how many days?  Days    Don’t know | | *(tick one appropriate answer, if yes then indicate number of days or tick don’t know if this is not known.)* |
|  | **Antimicrobial therapy** |  | |  |
| 9.1 | Were **first-line** antibiotic(s) given? | Yes  No (none noted)  Unclear from notes | | *(tick one appropriate answer.)*  **If no skip to 9.13** |
| 9.2 | **First-line** antibiotic 1 | Name: _______________  Individual dose:  Dose units:  micrograms  milligrams  Other, specify: ____________  Total number of doses given: | | *(If given indicate the name, individual dose, dose units and total number of doses given.)* |
| 9.3 | **First-line** antibiotic 2 | Name: _______________  Individual dose:  Dose units:  micrograms  milligrams  Other, specify: ____________  Total number of doses given: | | *(If given indicate the name, individual dose, dose units and total number of doses given.)* |
| 9.4 | **First-line** antibiotic 3 | Name: _______________  Individual dose:  Dose units:  micrograms  milligrams  Other, specify: ____________  Total number of doses given: | | *(If given indicate the name, individual dose, dose units and total number of doses given.)* |
| 9.5 | Were **second-line** antibiotic(s) given? | Yes  No (none noted)  Unclear from notes | | *(tick one appropriate answer.)*  **If no skip to 9.13** |
| 9.6 | **Second-line** antibiotic 1 | Name: _______________  Individual dose:  Dose units:  micrograms  milligrams  Other, specify: ____________  Total number of doses given: | | *(If given indicate the name, individual dose, dose units and total number of doses given.)* |
| 9.7 | **Second-line** antibiotic 2 | Name: _______________  Individual dose:  Dose units:  micrograms  milligrams  Other, specify: ____________  Total number of doses given: | | *(If given indicate the name, individual dose, dose units and total number of doses given.)* |
| 9.8 | **Second-line** antibiotic 3 | Name: _______________  Individual dose:  Dose units:  micrograms  milligrams  Other, specify: ____________  Total number of doses given: | | *(If given indicate the name, individual dose, dose units and total number of doses given.)* |
| 9.9 | Were **third-line** antibiotic(s) given? | Yes  No (none noted)  Unclear from notes | | *(tick one appropriate answer.)*  **If no skip to 9.13** |
| 9.10 | **Third-line** antibiotic 1 | Name: _______________  Individual dose:  Dose units:  micrograms  milligrams  Other, specify: ____________  Total number of doses given: | | *(If given indicate the name, individual dose, dose units and total number of doses given.)* |
| 9.11 | **Third-line** antibiotic 2 | Name: _______________  Individual dose:  Dose units:  micrograms  milligrams  Other, specify: ____________  Total number of doses given: | | *(If given indicate the name, individual dose, dose units and total number of doses given.)* |
| 9.12 | **Third-line** antibiotic 3 | Name: _______________  Individual dose:  Dose units:  micrograms  milligrams  Other, specify: ____________  Total number of doses given: | | *(If given indicate the name, individual dose, dose units and total number of doses given.)* |
| 9.13 | Was antiviral given? | Yes  No (none noted)  Unclear from notes | | *(tick one appropriate answer.)*  **If no skip to 9.15** |
| 9.14 | Antiviral | Name: _______________  Individual dose:  Dose units:  micrograms  milligrams  Other, specify: ____________  Total number of doses given: | | *(If given indicate the name, individual dose, dose units and total number of doses given.)* |
| 9.15 | Was antifungal given? | Yes  No (none noted)  Unclear from notes | | *(tick one appropriate answer.)*  **If no skip to 10.1** |
| 9.16 | Antifungal | Name: _______________  Individual dose:  Dose units:  micrograms  milligrams  Other, specify: ____________  Total number of doses given: | | *(If given indicate the name, individual dose, dose units and total number of doses given.)* |
|  | **Anticonvulsant therapy** |  | |  |
| 10.1 | Was anticonvulsant therapy given? | Yes  No (none noted)  Unclear from notes | | *(tick one appropriate answer.)*  **If no skip to 11.1** |
| 10.2 | Anticonvulsant 1 | Name: _______________  Individual dose:  Dose units:  micrograms  milligrams  Other, specify: ____________  Total number of doses given: | | *(If given indicate the name, individual dose, dose units and total number of doses given.)* |
| 10.3 | Anticonvulsant 2 | Name: _______________  Individual dose:  Dose units:  micrograms  milligrams  Other, specify: ____________  Total number of doses given: | | *(If given indicate the name, individual dose, dose units and total number of doses given.)* |
|  | **Other drug therapies** |  | |  |
| 11.1 | Was an inotrope given? | Yes  No (none noted)  Unclear from notes | | *(tick one appropriate answer.)*  **If no skip to 11.4** |
| 11.2 | Inotrope 1 | Name: _______________  Individual dose:  Dose units:  micrograms  milligrams  Other, specify: ____________  Total number of doses given: | | *(If given indicate the name, individual dose, dose units and total number of doses given.)* |
| 11.3 | Inotrope 2 |  | | *If given indicate the name, individual dose, dose units and total number of doses given.)* |
| 11.4 | Was surfactant given? | Yes  No (none noted)  Unclear from notes | | *(tick one appropriate answer.)*  **If no skip to 11.6** |
| 11.5 | Surfactant | Name: _______________  Individual dose:  Dose units:  micrograms  milligrams  Other, specify: ____________  Total number of doses given: | | *(If given indicate the name, individual dose, dose units and total number of doses given.)* |
| 11.6 | Were any other drugs given? | Yes  No (none noted)  Unclear from notes | | *(tick one appropriate answer.)*  **If no skip to 12.1** |
| 11.7 | Drug 1 | Name: _______________  Individual dose:  Dose units:  micrograms  milligrams  Other, specify: ____________  Total number of doses given: | | *If given indicate the name, individual dose, dose units and total number of doses given.)* |
| 11.8 | Drug 2 | Name: _______________  Individual dose:  Dose units:  micrograms  milligrams  Other, specify: ____________  Total number of doses given: | | *If given indicate the name, individual dose, dose units and total number of doses given.)* |
| 11.9 | Drug 3 | \| Name: _______________  Individual dose:  Dose units:  micrograms  milligrams  Other, specify: ____________  Total number of doses given: \| \| --- \| | | *If given indicate the name, individual dose, dose units and total number of doses given.)* |
|  | **Surgical interventions** |  | |  |
| 12.1 | Was any neuro-surgical intervention performed? e.g. Burr hole, CNS (VP) shunt insertion. | Yes  No (none noted)  Unclear from notes | | *(tick one appropriate answer.)*  **If no skip to 12.3** |
| 12.2 | If yes, please provide a short description of the surgical procedures performed. | Free text | |  |
| 12.3 | Was any other surgical intervention performed? | Yes  No (none noted)  Unclear from notes | | *(tick one appropriate answer.)*  **If no skip to 13.1** |
| 12.4 | If yes, please provide a short description of the surgical procedures performed. | Free text | |  |
|  | **Lab tests** |  | |  |
| 13.1 | Were any laboratory diagnostic tests performed? | Yes  No (none noted)  Unclear from notes  If yes, specify how many. | | *(tick one appropriate answer, if yes then indicate the number of tests performed)*  **If no skip to 14.1** |
| 13.2 | Was blood culture performed? | Yes  No (none noted)  Unclear from notes  If yes, specify how many. | | *(tick one appropriate answer, if yes then indicate the number of tests performed)* |
| 13.3 | Were molecular assays performed? | Yes  No (none noted)  Unclear from notes  If yes, specify how many. | | *(tick one appropriate answer, if yes then indicate the number of tests performed)* |
| 13.4 | Was complete / full blood count performed? | Yes  No (none noted)  Unclear from notes  If yes, specify how many. | | *(tick one appropriate answer, if yes then indicate the number of tests performed)* |
| 13.5 | Was blood biochemistry performed (renal and/or liver function) | Yes  No (none noted)  Unclear from notes  If yes, specify how many. | | *(tick one appropriate answer, if yes then indicate the number of tests performed)* |
| 13.6 | Was serum bilirubin performed? | Yes  No (none noted)  Unclear from notes  If yes, specify how many. | | *(tick one appropriate answer, if yes then indicate the number of tests performed)* |
| 13.7 | Was blood glucose test performed? | Yes  No (none noted)  Unclear from notes  If yes, specify how many. | | *(tick one appropriate answer, if yes then indicate the number of tests performed)* |
| 13.8 | Was lumbar puncture performed? | Yes  No (none noted)  Unclear from notes  If yes, specify how many. | | *(tick one appropriate answer, if yes then indicate the number of tests performed)* |
| 13.9 | Was CSF culture performed? | Yes  No (none noted)  Unclear from notes  If yes, specify how many. | | *(tick one appropriate answer, if yes then indicate the number of tests performed)* |
| 13.10 | Was urine culture performed? | Yes  No (none noted)  Unclear from notes  If yes, specify how many. | | *(tick one appropriate answer, if yes then indicate the number of tests performed)* |
|  | **Diagnostic tests** |  | |  |
| 14.1 | Was a hearing assessment performed? | Yes  No (none noted)  Unclear from notes  If yes, please give details of type of test performed _____________________________ | | *(tick one appropriate answer, if yes then provide details of the tests performed)* |
| 14.2 | Was a vision assessment performed | Yes  No (none noted)  Unclear from notes  If yes, please give details of type of test performed _____________________________ | | *(tick one appropriate answer, if yes then provide details of the tests performed)* |
| 14.3 | Was EEG monitoring performed? | No  Yes, single  Yes, continuous  Unclear from notes  If continuous, specify for number of days. | | *(tick one appropriate answer, if continuous monitoring then indicate number of days.)* |
| 14.4 | Was an electrocardiogram performed? | No  Yes  Unclear from notes  If yes, specify how many | | *(tick one appropriate answer, if yes then indicate the number of tests performed)* |
|  | **Diagnostic imaging** |  | |  |
| 15.1 | Was diagnostic imaging performed? | Yes  No (none noted)  Unclear from notes | | **If no skip to end** |
| 15.2 | Was an x-ray performed | No  Yes  Unclear from notes  If yes, specify how many | | *(tick one appropriate answer, if yes then indicate the number of tests performed)* |
| 15.3 | Was an ultrasound performed? | No  Yes  Unclear from notes  If yes, specify how many | | *(tick one appropriate answer, if yes then indicate the number of tests performed)* |
| 15.4 | Was a CT performed? | No  Yes  Unclear from notes  If yes, specify how many | | *(tick one appropriate answer, if yes then indicate the number of tests performed)* |
| 15.5 | Was an MRI performed? | No  Yes  Unclear from notes  If yes, specify how many | | *(tick one appropriate answer, if yes then indicate the number of tests performed)* |
| 15.6 | Was an echocardiogram performed? | No  Yes  Unclear from notes  If yes, specify how many | | *(tick one appropriate answer, if yes then indicate the number of tests performed)* |
| 15.7 | Was any other imaging procedure performed? | Yes  No (none noted)  Unclear from notes  If yes, please give details of type of  test performed _____________________________ | | *(tick one appropriate answer, if yes then provide details of the tests performed)* |

| Name: _______________  Individual dose:  Dose units:  micrograms  milligrams  Other, specify: ____________  Total number of doses given: | *(If given indicate the name, individual dose, dose units and total number of doses given.)* |
| --- | --- |

SECTION 3 – RESEARCHER FEEDBACK

**This section is to be completed by the researcher**

|  | **Data extraction** |  |  |
| --- | --- | --- | --- |
| 16.1 | Where there any difficulties in extracting data from the patient records? (e.g. difficulty reading illegible writing.) | Yes  No  If yes, please provide details:  _____________________________ | *(tick one appropriate answer, if yes then provide details of any problems that you faced )* |

**End**

# Supplementary Acute Data Collection Tool 3: Hospital Health Unit Costs

Hospital healthcare unit cost tool to assess economic impact of acute neonatal GBS disease

This document sets out the data on unit costs of acute hospital care that to be provided by hospitals. Separate questionnaires are used to capture healthcare resource use for individual participants, and the costs and economic impact from the household perspective.

SECTION 1 – STUDY INFORMATION

| **ID** | **Question / Data Item** | **Response Options** | **Comments** |
| --- | --- | --- | --- |
|  | **Study information** |  |  |
| 1.1 | Study Site | Free text |  |
| 1.2 | Country code | Argentina (01) / India (02) / Kenya (03) / Mozambique (04) / South Africa (05) |  |
| 1.3 | Clinic/Hospital code | Free text |  |
| 1.4 | Researcher ID | Free text |  |

SECTION 2 – HEALTHCARE RESOURCE UNIT COSTS

Estimated unit costs for each type of healthcare resource should be recorded in this section. Costs should ideally represent the economic cost (opportunity cost) of providing each resource unit. The source and methodology for each resource unit should be indicated e.g. top-down costing, micro-costing, existing prices / tariffs, market prices.

Cost estimates should avoid double-counting. For example, if the estimated cost per bed day for paediatric intensive care includes the cost of NG tubes, Oxygen, mechanical ventilation, etc then unit costs should not be recorded separately for these items.

| **ID** | **Resource / Data Item** | **Cost per unit (local currency)** | **Source / Methodology** |
| --- | --- | --- | --- |
|  | **Length of Stay** |  |  |
|  | Hospital bed (average across all beds.) | Cost per bed day |  |
|  | Paediatric intensive care bed. | Cost per bed day |  |
|  | Special care bed. | Cost per bed day |  |
|  | Normal paediatric bed. | Cost per bed day |  |
|  | **Supportive Care** |  |  |
|  | Cost of NG feeding tube | Cost per case |  |
|  | Cost of NG feeding tube | Cost per day |  |
|  | IV fluid | Cost per day |  |
|  | Oxygen | Cost per day |  |
|  | Mechanical ventilation | Cost per day |  |
|  | Dialysis | Cost per cay |  |
|  | **Antibiotic therapy** |  |  |
|  | Antibiotic **Name 1 (specify)** | Cost per dose mg/mL |  |
|  | Antibiotic **Name 2 (specify)** | Cost per dose mg/mL |  |
|  | Antibiotic **Name 3 (specify)** | Cost per dose mg/mL |  |
|  | Antibiotic **Name 4 (specify)** | Cost per dose mg/mL |  |
|  | Antibiotic **Name 5 (specify)** | Cost per dose mg/mL |  |
|  | Antibiotic **Name 6 (specify)** | Cost per dose mg/mL |  |
|  | **Lab tests** |  |  |
|  | Blood culture | Cost per test |  |
|  | Full blood count test | Cost per test |  |
|  | Blood WBC test | Cost per test |  |
|  | Serum bilirubin test | Cost per test |  |
|  | Blood glucose test | Cost per test |  |
|  | Lumbar puncture | Cost per procedure |  |
|  | CSF culture | Cost per test |  |
|  | **Diagnostic imaging** |  |  |
|  | X-ray | Cost per x-ray |  |
|  | Ultrasound scan | Cost per ultrasound scan |  |
|  | CT scan | Cost per CT scan |  |
|  | MRI scan | Cost per MRI scan |  |

| Supplementary Table A1: Descriptive statistics of the patients with bacterial sepsis and/or meningitis including only microbiological cases confirmed cases. | | | |
| --- | --- | --- | --- |
|  | **Mozambique**  **N (%)** | **South Africa**  **N (%)** | **Total**  **N (%)** |
| **Total** | **6** | **18** | **24** |
| **Sex** |  |  |  |
| Male | 2 (33%) | 12 (67%) | 14 (58%) |
| Female | 4 (67%) | 6 (33%) | 10 (42%) |
| **Day of life on admission** |  |  |  |
| 0-6 days | 4 (67%) | 6 (33%) | 10 (42%) |
| 7-14 days | 2 (33%) | 8 (44%) | 10 (42%) |
| 15-28 days | 0 (%) | 4 (22%) | 4 (17%) |
| **Place of delivery** |  |  |  |
| Home | 0 (0 %) | 0 (0%) | 0 (0%) |
| Hospital | 6 (100%) | 18 (100%) | 24 (100%) |
| **Gestational age (weeks)** |  |  |  |
| 32-34 weeks | 0 (0%) | 2 (11%) | 2 (8%) |
| 35-37 weeks | 2 (33%) | 6 (33%) | 8 (33%) |
| >37 weeks | 2 (33%) | 10 (56%) | 12 (50%) |
| Unknown | 2 (33%) | 0 (0%) | 2 (8%) |
| **Admitted at birth** |  |  |  |
| Yes | 3 (50%) | 0 (0%) | 3 (13%) |
| No | 3 (50%) | 18 (100%) | 21 (88%) |
| **Birth weight (grams)** |  |  |  |
| <1500 | 0 (0%) | 0 (0%) | 0 (0%) |
| 1500-2500 | 3 (50%) | 5 (28%) | 8 (33%) |
| 2501-3000 | 0 (0%) | 7 (39%) | 7 (29%) |
| > 3000 | 3 (50%) | 6 (33%) | 9 (38%) |
| **Syndrome** | | | |
| Meningitis | 0 (0%) | 2 (11%) | 2 (8%) |
| Sepsis | 6 (100%) | 16 (89%) | 22 (92%) |
| **Bacteria detected** |  |  |  |
| Group B Streptococcus | 1 (%) | 8 (44%) | 8 (33%) |
| *Escherichia coli* | 0 (%) | 6 (33%) | 6 (25%) |
| *Staphylococcus aureus* | 0 (%) | 4 (22%) | 4 (17%) |
| *Gram negative Rod* | 5 (83%) | 0 (0%) | 5 (21%) |
| **Length of stay (days)** |  |  |  |
| 0-7 days | 2 (33%) | 0 (0%) | 2 (8%) |
| 8-14 days | 3 (50%) | 6 (33%) | 9 (38%) |
| >15 days | 1 (17%) | 12 (67%) | 13 (54%) |
| **Discharge status** |  |  |  |
| Discharged alive | 5 (83%) | 17 (94%) | 22 (92%) |
| Referred to another hospital | 1 (17%) | 0 (0%) | 1 (4%) |
| Died | 0 (0 %) | 1 (6%) | 1 (4%) |
| *Legend: Percentage may not sum up to 100% due to rounding* | | | |

| Supplementary Table A2: Descriptive analysis of cost data for neonatal sepsis and/or meningitis inpatients (International Dollars 2019) including only microbiologically confirmed cases. | | | | | | | |
| --- | --- | --- | --- | --- | --- | --- | --- |
| **Hospitalization costs** | | | | | | | |
|  | **Mozambique (N=6)** | | | | **South Africa (N=18)** | | |
|  | **Mean** | **Median** | | **IQR** | **Mean** | **Median** | **IQR** |
| **Hospital stay** (pediatric / intensive care bed) | 140.02 | 137.56 | | 103.17-182.27 | 379.41 | 349.62 | 308.83-419.55 |
| **Supportive care** |  |  | |  |  |  |  |
| Oral feeding | 16.99 | 0.00 | | 0.00-0.00 | Included in the hospital daily rate | | |
| NG feeding | / |  | |  | Included in the hospital daily rate | | |
| IV fluid | / |  | |  | Included in the hospital daily rate | | |
| Parenteral feeding | / |  | |  | Included in the hospital daily rate | | |
| Nasal cannula oxygen | / |  | |  | Included in the hospital daily rate | | |
| Phototherapy | / |  | |  | Included in the hospital daily rate | | |
| Hypothermia | Data not available | | | | / | | |
| **Antimicrobial therapy** |  |  | |  |  |  |  |
| Antibiotics (first line) | 66.95 | 78.77 | | 78.77-78.77 | 25.29 | 14.19 | 7.02-45.28 |
| Antibiotics (second line) | 6.94 | 0.00 | | 0.00-0.00 | 14.04 | 2.93 | 0.00-17.26 |
| Antibiotics (third line) | / |  | |  | 0.89 | 0.00 | 0.00-0.00 |
| Antifungals | / |  | |  | 0.64 | 0.00 | 0.00-0.00 |
| **Anticonvulsants** | / |  | |  | 2.27 | 0.00 | 0.00-0.00 |
| **Other drugs** | 0.85 | 0.85 | | 0.00-0.00 | 2.45 | 0.00 | 0.00-5.02 |
| **Laboratory test** |  |  | |  |  |  |  |
| Blood culture | 41.71 | 41.77 | | 0.00-83.43 | 19.20 | 16.08 | 16.08-24.12 |
| Lumbar puncture | 60.59 | 60.59 | | 0.00-121.19 | 27.65 | 23.70 | 23-70-23.70 |
| CSF culture | 48.86 | 48.86 | | 0.00-97.72 | 17.26 | 14.79 | 14.79-14.79 |
| Urine culture | 11.08 | 0.00 | | 0.00-0.00 | 9.99 | 8.99 | 8.99-8.99 |
| Full blood count test | 4.99 | 0.00 | | 0.00-0.00 | 18.32 | 18.32 | 9.16-27.48 |
| Blood biochemistry | / |  | |  | 81.00 | 36.45 | 36.45-100.24 |
| Serum bilirubin test | / |  | |  | 5.46 | 0.00 | 0.00-5.79 |
| Blood glucose test | 5.42 | 0.00 | | 0.00-12.20 | 12.25 | 4.79 | 0.00-4.79 |
| **Diagnostic assessment** |  |  | |  |  |  |  |
| Hearing assessment | / |  | |  | Included in the hospital daily rate | | |
| Electrocardiogram | / |  | |  | Included in the hospital daily rate | | |
| **Diagnostic imaging** |  |  | |  |  |  |  |
| X-ray | / |  | |  | 4.18 | 0.00 | 0.00-7.52 |
| Ultrasound scan | / |  | |  | 13.03 | 0.00 | 0.00-19.55 |
| Echocardiogram | / |  | |  | 50.13 | 0.00 | 0.00-0.00 |
| **TOTAL** | **413.41** | **386.12** | | **221.48-514.39** | **684.35** | **653.92** | **543.64-827.83** |
| **Household expenses** | | | | | | | |
|  | **Mozambique (N=4)**** | | | | **South Africa (N=18)** | | |
|  | **Mean** | **Median** | **IQR** | | **Mean** | **Median** | **IQR** |
| Healthcare | 0 | 0.00 | 0.00-0.00 | | 10.03 | 0.00 | 0.00 |
| Transportation | 8.70 | 8.91 | 5.95-11.46 | | 42.28 | 30.83 | 19.25-70.38 |
| Food and accommodation | 0.00 | 0.00 | 0.00-0.00 | | 0.00 | 0.00 | 0.00-0.00 |
| **TOTAL** | **8.70** | **8.92** | **5.95-11.46** | | **52.31** | **30.82** | **19.25-73.08** |
| **based on one estimate in Mozambique;*** less than 6 due to missing values in the cost of transportation  *Legend: IQR=interquartile range; “/”=does not apply/service not provided to the neonate* | | | | | | | |
